# Effects of a novel curcumin derivative on the functions of kidney in streptozotocin-induced type 2 diabetic rats

**DOI:** 10.1007/s10787-018-0449-1

**Published:** 2018-03-26

**Authors:** Xuegu Xu, Yonghao Cai, Yinfei Yu

**Affiliations:** Department of Pharmacy, The Eye Hospital of Wenzhou Medical University, Wenzhou, 325003 Zhejiang China

**Keywords:** Diabetic nephropathy, Curcumin derivative, B6, Hyperglycemia

## Abstract

**Objective:**

B6, an analog of curcumin, is a compound isolated from a traditional Chinese medicine Turmeric. In this paper, we aimed to explore the efficacy of B6 on diabetic nephropathy and the related mechanisms.

**Materials and methods:**

The effects of B6 were studied on fast-blood glucose, serum creatinine, urea nitrogen, urine albumen/24 h, pathological changes of main organs, the levels of ACE2 and ACE2 mRNA in the rat model of diabetes induced by streptozotocin.

**Results:**

The results showed that B6 treatment could reduce serum creatinine, urea nitrogen, urine albumen/24 h, decrease the level of AngII, improve the renal pathological changes in diabetic rats and increase the levels of ACE2 and ACE2 mRNA.

**Conclusion:**

These results suggested B6 could protect the renal function of diabetic rats. This study provided scientific basis for the further researches and clinical applications of B6.

## Introduction

Diabetic nephropathy (DN) is a common complication due to diabetes and the most common cause of end-stage renal disease (Kanwar et al. 2011). It has a strong impact on patients’ qualities of life and survival rate. Therefore, studies of the treatment of DN have representative and guiding meanings.

Curcumin (1, 7-bis-(4-hydroxy-3-methoxyphenyl)-1, 6-heptadiene-3, 5-dione) is the main active component of the natural turmeric (Bengmark 2006). It has been extensively demonstrated as a multifunctional agent on cancer, inflammation, cholesterol-lowering and inflammatory-related diseases (Di et al. 2010; Shu et al. 2007; Sun et al. 2014). It showed that curcumin could significantly improve the renal functions of streptozotocin-induced diabetic rats. But the exact mechanisms remained to be further explained (Tikoo et al. 2008). However, the clinical applications of curcumin have been significantly limited by its low water solubility, short half-life in plasma and poor bioavailability (Pan et al. 2012; Epstein et al. 2010). Modifying the chemical structure of curcumin is one approach to overcome the poor bioavailability or further enhance its effects. A novel curcumin analogue, (E, E)-1, 5-bis(2-bromophenyl)-1,4-pentadiene-3-one (B6), was identified as a high-active curcumin analogue screening from a series of new synthetic curcumin derivatives in vitro. The activities of curcumin and synthetic derivatives in vitro were mainly detected through the inhibition level of 11βHSD1 and 11βHSD2 of humans and rats. The results showed that B6 can strongly inhibit 11βHSD1 and 11βHSD2 in humans and rats. We set up B6 HPLC determination method in rat plasma and studied its pharmacokinetics in rats. The results showed the bioavailability of gastrointestinal tract in rats was 8.884 ± 0.879% and the plasma half-life was 143.535 ± 48.375 min (Jiang et al. 2016).

In this study, we showed that B6 could reduce the levels of some biochemical indexes, improve the renal pathological changes, enhance the level of ACE2 and prevent renal injury in experimental diabetic rats.

## Materials and methods

### Drugs and chemicals

**Fig. 1:**
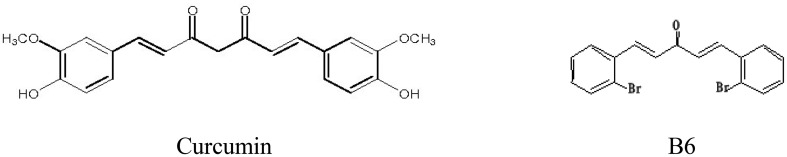
Chemical structures of curcumin and B6

Compound B6 was synthesized and characterized by Wenzhou Medical University. A high-performance liquid chromatography method was used to determine its purity (98.67%). The structure of B6 is shown in Fig. 1. B6 was dissolved in corn oil for in vivo experiments. Streptozotocin (STZ) was purchased from Sigma-Aldrich (St. Louis, MO, USA).

### Animal experiments

Protocols for animal studies were approved by the Wenzhou Medical University Animal Policy and Welfare Committee (Approved documents: 2009/APWC/0031). Fifity-eight male Wistar rats (160–180 g) were obtained from the Animal Center of Wenzhou Medical University (Wenzhou, China). Animals were housed at 21–25 °C with a 12:12 h light/dark cycle. Water and a standard diet were consumed. After 1 week, 58 Wistar rats were divided into two groups randomly: (1) the normal control group (NC Group, 10 Wistar rats); (2) high-fat- and high-sugar group (Model Group, 48 Wistar rats). The composition of high-sugar- and high-fat diet for Wistar rats was as follows: 68% common diet, 20% sucrose, 10% lard oil and 2% cholesterol. The obesity rat model can be built by feeding high-sugar- and high-fat diet for 4 weeks. After 4 weeks, the 48 Wistar rats were treated with intraperitoneal injection of STZ (30 mg/kg dissolved in citrate buffer, pH = 4.5) to induce type 2 diabetes. The control group (10 Wistar rats) was intraperitoneally treated with the same volume of citrate buffer. The fasting-blood glucose was monitored using a glucometer (ACCU produced by Roche) on days 3 and 7 after STZ injection. The fasting-blood glucose > 8 mmol/l and random-blood glucose > 16.7 mmol/l were considered diabetic, then the 48 rats were randomly divided into four groups: (1) DM group (*n* = 12), (2) B6 low-dose group [B6(L), *n* = 12], (3) B6 middle-dose group [B6(M), *n* = 12], (4) B6 high-dose group [B6(H), *n* = 12]. The dosages of B6 were 1, 3 and 9 mg/kg days, respectively. NC group and DM group were gastrointestinally administrated the same volume of corn oil. After 8 weeks, animals were sacrificed under 10% chloral hydrate anesthesia. Venous blood was taken after anesthesia. Kidney tissues were embedded in 4% paraformaldehyde for pathological analysis or were frozen in liquid nitrogen for gene and protein expression analysis.

### Determination of fast-blood glucose, serum creatinine, serum urea nitrogen and urine protein/24 h

The levels of fasting-blood glucose, creatinine and urea nitrogen were determined by 7600-120ISE biochemical analyzer (Hitachi Limited, Tokyo, Japan) and the level of urine protein was determined by Coomassie brilliant blue staining according to the manufacturer’s instructions.

### Determination of serum AngII and serum insulin

The levels of serum AngII and insulin were measured by radioimmunoassay. Radioimmunoassay kit of serum AngII and insulin were purchased from Beijing SINO-UK Institute of Biotechnology (Beijing, China) and Northern Beijing Institute of Biotechnology (Beijing, China), respectively. The testing steps referred to the instructions of kit.

### Histopathology

Kidneys were soaked in 4% paraformaldehyde solution for pathological analysis, embedded in paraffin and sectioned at 5μm. After dehydration, sections were stained with haematoxylin and eosin. To evaluate the histopathological damage, each image of sections was examined by a light microscope (Olympus, Tokyo, Japan).

### Immunohistochemical assay of ACE2

Immunohistochemistry was performed on paraffin sections (5μm) using a microwave-based antigen retrieval technique. The slides were incubated overnight at 4 °C in a humidified chamber with NUCB2 rabbit anti-rat polyclonal antibody (1:100) (from Santa Cruz Biotechnology, Inc., Dallas, TX, USA) antibodies. The next day, each slice was added with a drop of non-biotinylated goat anti-rabbit polyperoxidase. After incubating for 30 min at room temperature, immunostaining was visualized with 0.05% diaminobenzidine. Sections were examined using a light microscope (Olympus, Tokyo, Japan) and imaged with a high-resolution camera at a magnification of × 400. The negative control was using PBS solution (pH 7.2–7.6) instead of NUCB2.

Each slice selected 5 fields of vision (× 400) randomly. Image-pro plus 5.0 was used to measure intergral optical density (IOD) of each slice. The greater of IOD value showed the higher level of the protein. The average value of 6 times of IOD was calculated as the IOD value of the slice.

### RT-PCR assay of ACE mRNA and ACE2 mRNA

Total RNA was isolated from kidney tissues using TRIZOL (Invitrogen, Carlsbad, CA, USA). DNA Marker I was purchased from Tiangen Biochemical Technology co., LTD (Beijing, China). Reverse transcription PCR were performed with an RT-PCR Kit (TaKaRa, Dalian, China). RT-PCR was carried out using the Eppendorf Realplex 4 instrument (Eppendorf, Hamburg, Germany).

### Statistical analysis

All the research data were processed by SPSS 19.0. The measurement data were expressed as the Mean ± SD. ANOVA and GraphPad Pro (GraphPad, San Diego, CA, USA) were used to analyse the statistical significance between sets of data. Differences were considered to be significant at *p* < 0.05.

## Results

### B6 administration increased diabetic rats’ body weight, but did not affect the ratio of kidney/body weight

After 8 weeks, there was a significant decline in mean body weight in DM group and B6 treatment groups. But there was a significant increase in B6(L) and B6(M) groups. However, there were no significant differences in kidney/body weight ratio in DM group and B6 treatment groups (Table 1).

**Table 1:** Comparisons of body weight, kidney weight and kidney/body weight ratio in different groups after 8 weeks ($\bar{x}$ ± s)

Groups	Number	Body weight (g)	Kidney weight (g)	1000 × kidney/body weight ratio
NC	10	350.00 ± 13.22	1.31 ± 0.21	3.75 ± 0.46
DM	12	228.33 ± 20.21**	1.27 ± 0.23	5.53 ± 0.60**
B6(L)	12	253.33 ± 15.71**^##^	1.23 ± 0.15	4.84 ± 0.32**
B6(M)	12	255.00 ± 16.07**^##^	1.24 ± 0.16	4.84 ± 0.40**
B6(H)	12	239.30 ± 10.58**	1.23 ± 0.13	5.15 ± 0.39**

Data were presented as means ± SD. *p < 0.05, **p < 0.01 vs. NC group; ^#^*p* < 0.05, ^##^*p* < 0.01 vs. DM group

### Administration of B6 improved the metabolic and histological abnormality

Table 2 showed that B6 treatment did not affect the blood glucose profile of diabetic rats after 8 weeks. The urea nitrogen, serum creatinine and urine protein/24 h increased significantly in DM relative to control. After treatment with B6 for 8 weeks, the urea nitrogen, serum creatinine and urine protein/24 h were significantly reduced, especially in B6(M) group.

**Table 2:** Comparisons of glucose, serum creatinine, urea nitrogen and urine albumen/24 h in different groups ($\bar{x}$ ± s)

Group	Blood glucose mmol/L)	Serum creatinine (μmol/L)	Urea nitrogen (mmol/L)	Urine albumen mg/24 h)
NC	8.33 ± 0.97	26.50 ± 2.74	7.51 ± 1.62	4.16 ± 1.13
DM	25.75 ± 3.60**	53.50 ± 4.23**	18.14 ± 2.33**	16.55 ± 3.16**
B6(L)	23.19 ± 6.37**	36.70 ± 2.26**^##^	15.72 ± 2.55**^#^	12.97 ± 2.99**^##^
B6(M)	28.37 ± 3.37**	32.50 ± 3.41**^##^	14.18 ± 1.38**^##^	13.13 ± 1.26**^#^
B6(H)	26.89 ± 3.94**	44.20 ± 3.68**^##^	17.78 ± 0.89**	13.22 ± 1.28**^#^

Data were presented as means ± SD. *p < 0.05, **p < 0.01 vs. NC group; ^#^*p* < 0.05, ^##^*p* < 0.01 vs. DM group

There were no significant differences of serum insulin values in DM group and B6 treatment groups (Table 3), indicating B6 treatment did not affect the serum insulin. Serum AngII increased significantly in DM group relative to control. But there was a significant decrease in serum AngII in B6(L) and B6(M) groups (Table 3).

**Table 3:** Comparisons of insulin and AngII in different groups ($\bar{x}$ ± s)

Group	Number	Insulin (mU/L)	AngII (pg/ml)
NC	10	25.46 ± 2.78	436.90 ± 51.01
DM	12	37.02 ± 4.36**	868.28 ± 73.15**
B6(L)	12	39.21 ± 4.00**	633.86 ± 46.49**^#^
B6(M)	12	37.55 ± 2.83**	555.26 ± 46.04*^##^
B6(H)	12	36.41 ± 1.75**	835.29 ± 98.05**

Data were presented as means ± SD. *P < 0.05, **p < 0.01 vs. NC group; ^#^*p* < 0.05, ^##^*p* < 0.01 vs. DM group

**Fig. 2:**
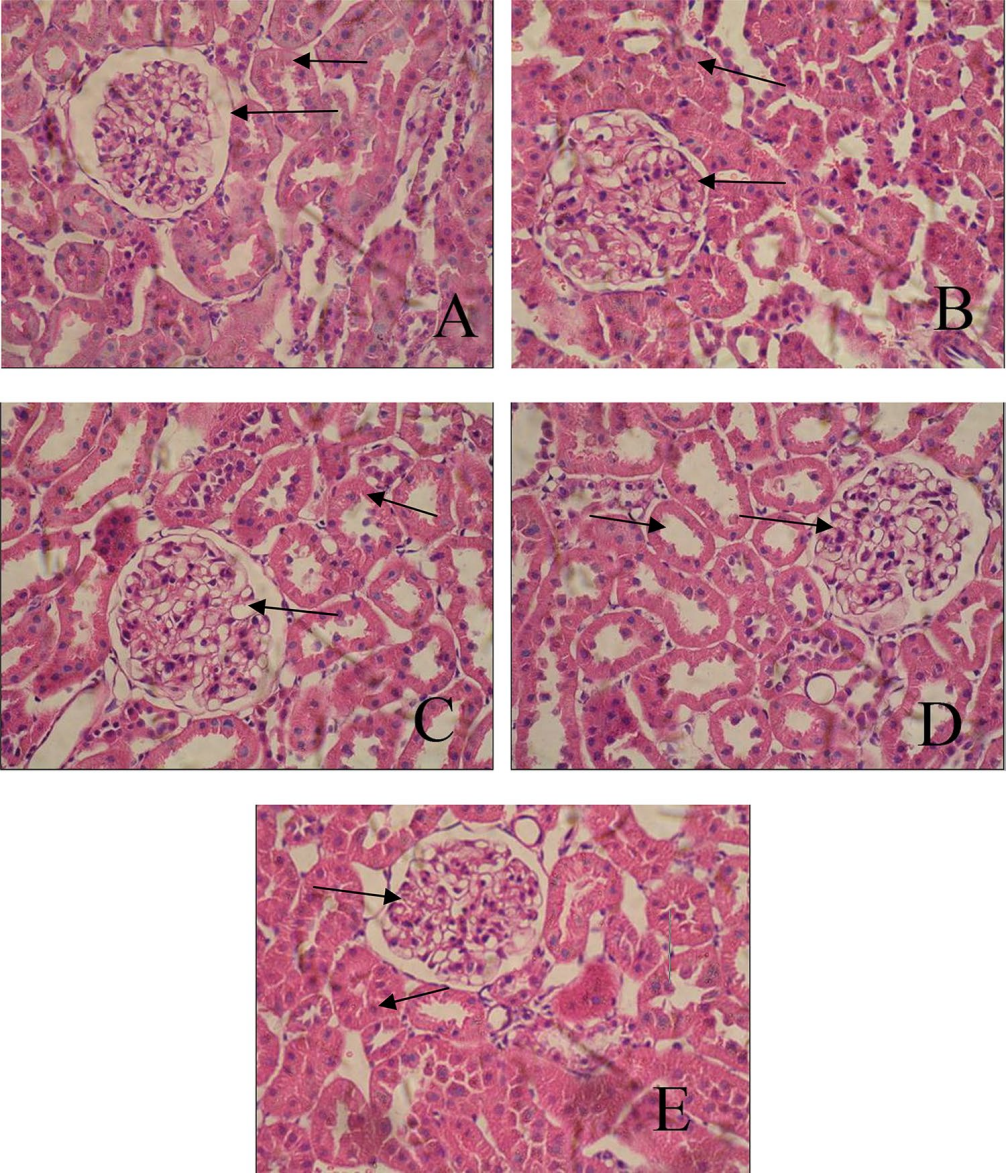
Histomorphological changes of renal tissues in different groups (HE, × 400) **a** NC group; **b**: DM group; **c** B6(L) group; **d** B6(M) group; **e** B6(H) group

**Fig. 3:**
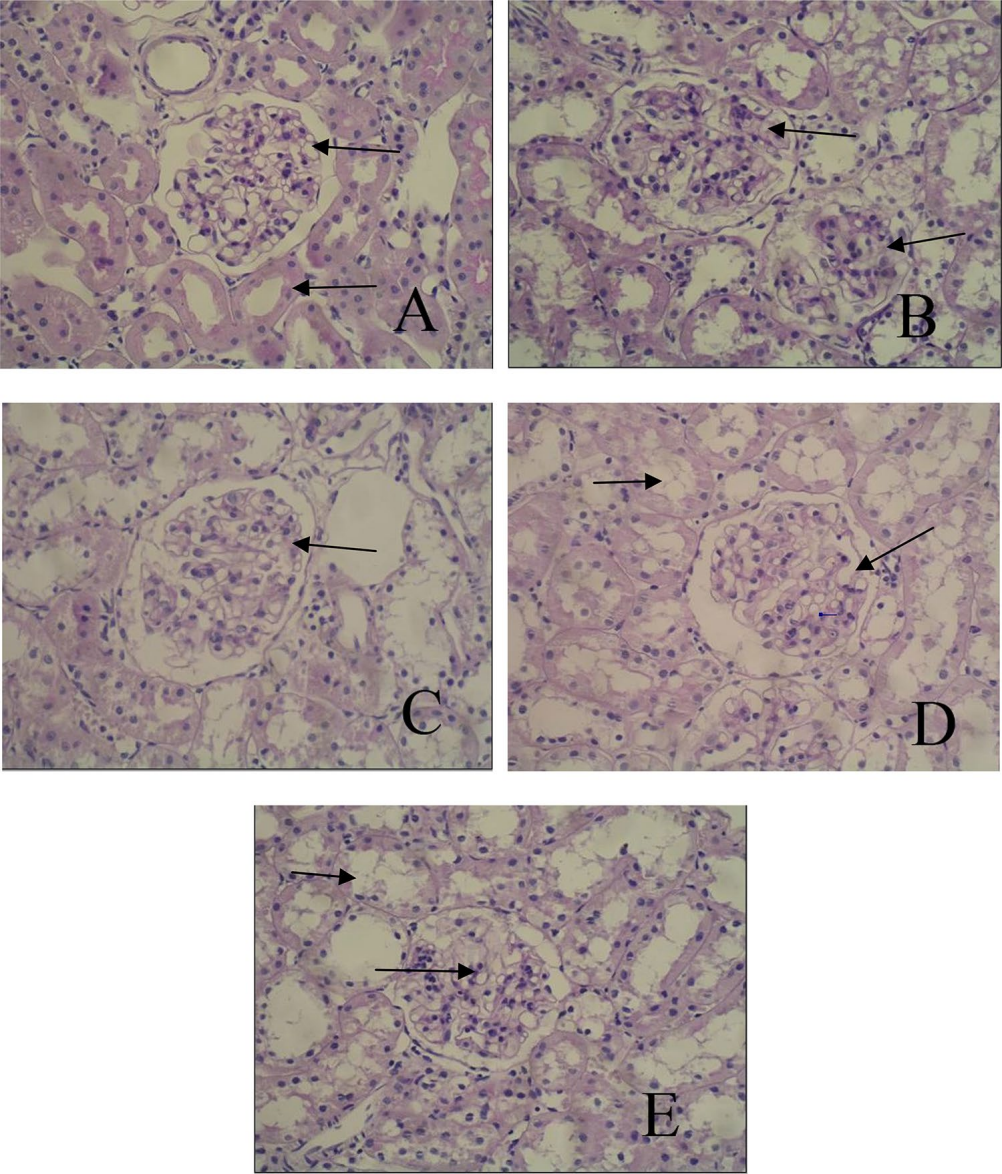
Histomorphological changes of renal tissues in different groups (PAS, × 400) **a** NC group; **b**: DM group; **c**: B6(L) group; **d**: B6(M) group; **e**: B6(H) group

The H&E staining (Fig. 2) revealed the glomerular hypertrophy, glomerulus with glomerulosclerosis and expansion, predominance of dense hyaline matrix and peripheral capillaries of thick stiff wall in the diabetic rats, whereas B6 treatment markedly ameliorated these diabetes-induced histopathological alterations. PAS further validated the histological renal improvements in B6 treatment groups when compared to that of untreated group. PAS staining (Fig. 3) demonstrated that B6 had a dose-dependent inhibition of diabetes-induced glycogen collection (purple plaques) in rats’ kidney, especially in B6(M) group.

### B6 administration did not affect the levels of ACE mRNA in diabetic rats, but upregulated the levels of ACE2 mRNA and ACE2

**Fig. 4:**
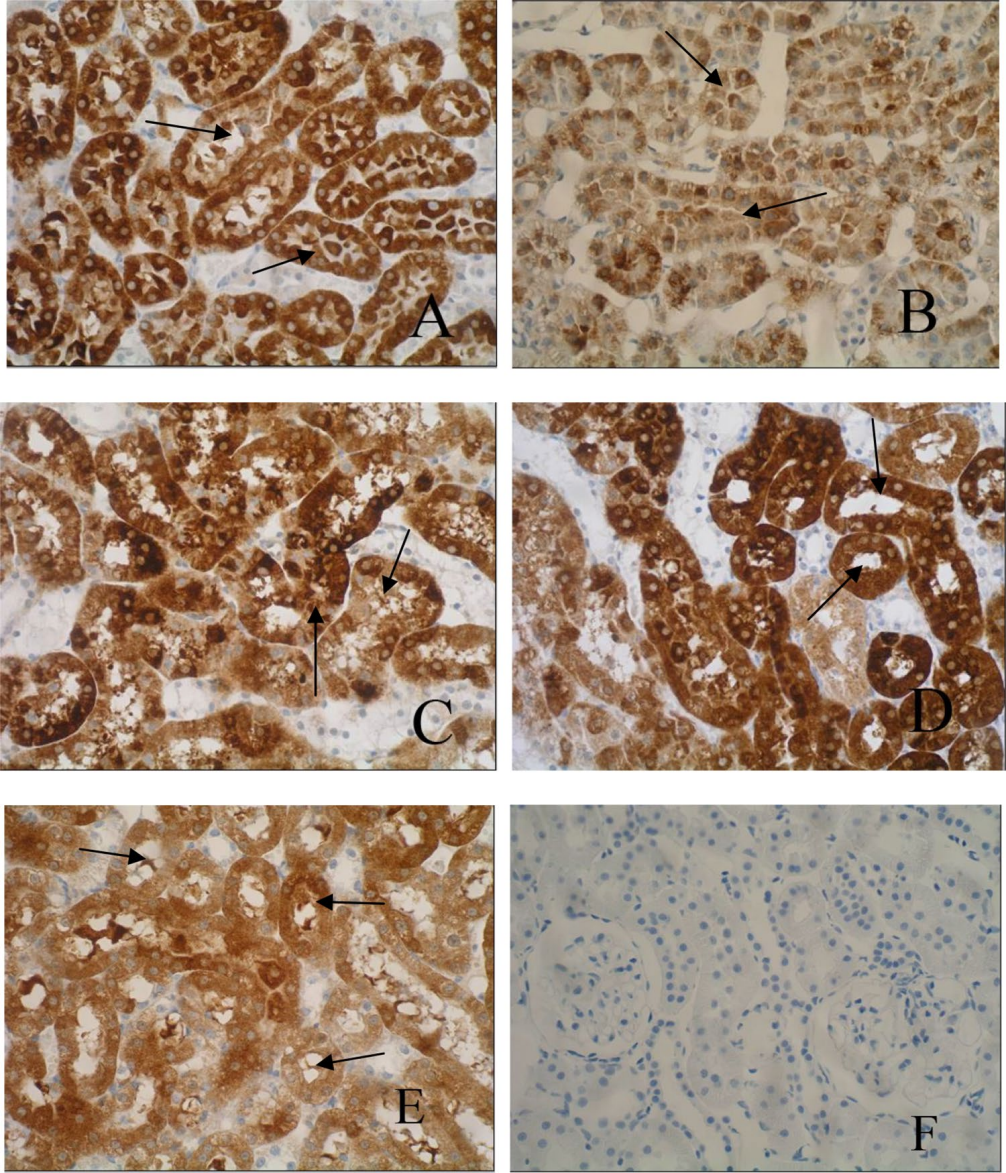
Expression of ACE2 in different groups(Immunohistochemistry, × 400) **a** NC group; **b** DM group; **c** B6(L) group; **d** B6(M) group; **e** B6(H) group; **f** negative control group

The balance between ACE and ACE2 is the key factor in regulating levels of angiotensin (Zhong et al. 2004; Song et al. 2013). Decreased expressions of ACE2 in kidney will result in an increase of AngII in local renal tubule, which can cause the renal interstitial fibrosis (Bürgelová et al. 2005). Immunohistochemistry staining was performed to observe the levels of ACE2. The levels of ACE2 significantly decreased in DM group after 8 weeks, compared with control group. But there was a significant increase in B6(L) and B6(M) groups (Table 4, Fig. 4).

**Table 4:** Comparisons of expression of ACE2 protein in different groups ($\bar{x}$ ± s)

Group	Number	Density (mean)
NC	5	0.388 ± 0.027
DM	6	0.238 ± 0.021**
B6(L)	6	0.333 ± 0.022**^##^
B6(M)	6	0.364 ± 0.036^##^
B6(H)	6	0.251 ± 0.021**

Data were presented as means ± SD. *p < 0.05, **p < 0.01 vs. NC group; ^#^*p* < 0.05, ^##^*p* < 0.01 vs. DM group

**Fig. 5:**
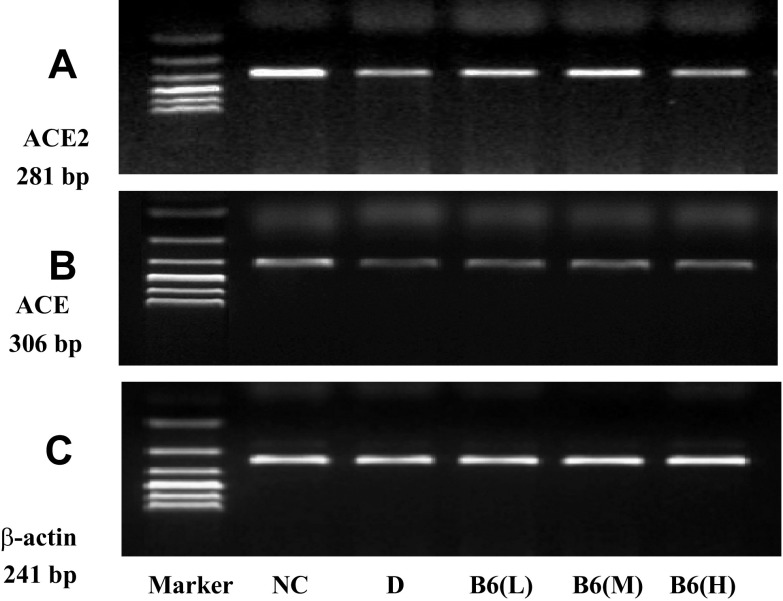
Levels of ACE2 mRNA, ACE mRNA and β-actin mRNA in different groups

RT-PCR was used to determine the relative content of ACE mRNA and ACE2 mRNA to validate whether B6 treatment had effects on the levels of ACE mRNA and ACE2 mRNA. The results showed that B6 treatment did not affect the relative content of ACE mRNA (Table 5, Fig. 5). There was a significant reduction in ACE2 mRNA in DM group relative to control. The relative content of ACE2 mRNA increased remarkably in B6(L) and B6(M) groups. And after the treatment of B6, the level of ACE2 in B6(M) group was the same with that in control group (Table 5, Fig. 5).

**Table 5:** Comparisons of relative content of ACE mRNA and ACE2 mRNA in different groups ($\bar{x}$ ± s)

Group	N	ACE/β-actin mRNA	ACE2/β-actin mRNA
NC	5	0.540 ± 0.022	0.577 ± 0.040
DM	6	0.437 ± 0.039**	0.387 ± 0.032**
B6(L)	6	0.422 ± 0.053*	0.458 ± 0.025**^#^
B6(M)	6	0.443 ± 0.023**	0.544 ± 0.072^#^
B6(H)	6	0.391 ± 0.048**	0.319 ± 0.051**

Data were presented as means ± SD. *p < 0.05, **p < 0.01 vs. NC group; ^#^*p* < 0.05, ^##^*p* < 0.01 vs. DM group

### Acute toxicity test of B6

Ten ICR mice were chosen randomly. Each mouse was intragastric administration at dose of B6 3 g/kg. After administration, ten ICR mice were given natural drink and diet. During 3 days of observation, no mouse died and all observation measures were normal. Acute toxicity test of mice showed B6 was safe.

## Discussion

The treatments of DN usually focus on insulin, angiotensin-converting enzyme inhibitors and angiotensin receptor antagonist. In this study, we demonstrated that a novel curcumin analogue, B6, efficiently attenuated diabetic renal injury via reductions of the serum creatinine, urea nitrogen and urine protein/24 h. Improvements of renal function in B6-treated groups can also be seen in histopathological changes.

Renin–angiotensin system (RAS) is a major contributor to systemic blood pressure. Recently, it has also been found as playing an important role in the development of DN (Carey and Siragy 2003). AngII is the main molecule of RAS, and relative with all pathological changes of DN, such as renal hemodynamic changes, accumulation of extracellular matrix, productions of the cytokines, podocyte injury, proteinuria and interstitial nephritis (Wolf 2004). Reduction of AngII can significantly improve the development of glomerular sclerosis and proteinuria (Ahmad et al. 1997). Our results showed AngII had a significant decrease in B6 treatment groups.

Angiotensinogen is broken down by renin to give angiotensin I (AngI), and angiotensin-converting enzyme (ACE) subsequently converts AngI to angiotensin II (AngII). In the kidneys, the ACE-AngII type 1 (AT1) axis (the classical RAS) promotes sodium and water retention, oxidative stress, vasoconstriction, cell proliferation, inflammation, and fibrosis. In 2000, ACE2 (Tipnis et al. 2000; Donoghue et al. 2000) was discovered, which indicated that the RAS is more complex than was previously imagined. ACE2 counteracts the effects of ACE by catabolizing AngII to produce Ang 1-7. The balance between the effects of these two molecules affects the renal RAS, and hence, the ACE/ACE2 ratio might represent the key parameter that is driving the regulation of the renal RAS (Bernardi et al. 2012; Ye et al. 2004; Wakahara et al. 2007; Soler et al. 2009; Batlle et al. 2012; Soler et al. 2013).

Therefore, ACE2 plays an important role in the development of renal diseases, such as diabetic nephropathy. It is suggested that the main priority of the RAS is to achieve an appropriate balance between ACE and ACE2 activity. The ACE-AngII-AT1 axis has been suggested to have detrimental effects on the RAS, whereas the ACE2-Ang 1-7-Mas axis counteracts the ACE-AngII-AT1 axis and seems to play a renoprotective role (Iwai and Horiuchi 2009). In a study involving diabetic rats, Tikellis et al. (2003) observed reduced renal expression levels of ACE and ACE2 mRNA, higher glomerular ACE and ACE2 protein expression levels, and lower tubular ACE and ACE2 protein expression levels at 24 weeks after the administration of STZ. In a study examining db/db mice without nephropathy, Ye et al. (2004) detected higher ACE2 protein expression and lower ACE protein expression in the animal’s renal tubules, which resulted in renoprotective effects. Moreover, they speculated that reduced ACE2 expression and upregulated ACE expression gradually induce kidney damage in diabetes (Ye et al. 2004).

Taken together, studies had suggested that upregulated ACE expression and downregulated ACE2 expression were seen at both the glomerular and tubular levels in established diabetic nephropathy.

The studies showed that there was a significant decrease of ACE in DM group and B6-treated groups relative to control, which may be related to the consumption of a large amount of ACE in the process of conversion from AngI to AngII. B6 did not affect on the activities of ACE in diabetic nephropathy rats, but could upregulate ACE2 and ACE2 mRNA, which regulated the balance of circulation and local blood vessels, maintained renal blood flow and reached the purpose of anti-renal fibrosis finally.

## Conclusion

Generally, the studies showed that B6 had positive effects on diabetic nephropathy. These results indicated that B6 was a new type of curcumin derivatives with great potential for development. We had also filtered other new curcumin derivatives, and only a few curcumin derivatives showed good characteristics. The absorption and metabolism of B6 was better than curcumin. And our laboratory study showed that B6 had an important role in regulating blood lipid and protecting fatty liver. Besides, no obvious acute toxic effects were observed. This study provided scientific basis for the further researches and clinical applications of B6.
